# New Estimates of Nitrogen Fixation on Early Earth

**DOI:** 10.3390/life14050601

**Published:** 2024-05-08

**Authors:** Madeline Christensen, Danica Adams, Michael L. Wong, Patrick Dunn, Yuk L. Yung

**Affiliations:** 1 Bellarmine Preparatory Marine Chemistry Program, Tacoma, WA 98405, USA; 2 Division of Geological and Planetary Sciences, California Institute of Technology, Pasadena, CA 91125, USA; 3 Department of Earth and Planetary Sciences, Harvard University, Cambridge, MA 02138, USA; 4 NHFP Sagan Fellow, NASA Hubble Fellowship Program, Space Telescope Science Institute, Baltimore, MD 21218, USA; 5 Earth and Planets Laboratory, Carnegie Institution for Science, Washington, DC 20015, USA; 6 Space Sciences Laboratory, University of California, Berkeley, CA 94720, USA; pdunn@ssl.berkeley.edu; 7 NASA Jet Propulsion Laboratory, Pasadena, CA 91109, USA

**Keywords:** astrobiology, origin of life, early Earth, photochemistry, atmospheric chemistry, nitrogen fixation, prebiotic chemistry

## Abstract

Fixed nitrogen species generated by the early Earth’s atmosphere are thought to be critical to the emergence of life and the sustenance of early metabolisms. A previous study estimated nitrogen fixation in the Hadean Earth’s N_2_/CO_2_-dominated atmosphere; however, that previous study only considered a limited chemical network that produces NO*_x_* species (i.e., no HCN formation) via the thermochemical dissociation of N_2_ and CO_2_ in lightning flashes, followed by photochemistry. Here, we present an updated model of nitrogen fixation on Hadean Earth. We use the Chemical Equilibrium with Applications (CEA) thermochemical model to estimate lightning-induced NO and HCN formation and an updated version of KINETICS, the 1-D Caltech/JPL photochemical model, to assess the photochemical production of fixed nitrogen species that rain out into the Earth’s early ocean. Our updated photochemical model contains hydrocarbon and nitrile chemistry, and we use a Geant4 simulation platform to consider nitrogen fixation stimulated by solar energetic particle deposition throughout the atmosphere. We study the impact of a novel reaction pathway for generating HCN via HCN_2_, inspired by the experimental results which suggest that reactions with CH radicals (from CH_4_ photolysis) may facilitate the incorporation of N into the molecular structure of aerosols. When the HCN_2_ reactions are added, we find that the HCN rainout rate rises by a factor of five in our 1-bar case and is about the same in our 2- and 12-bar cases. Finally, we estimate the equilibrium concentration of fixed nitrogen species under a kinetic steady state in the Hadean ocean, considering loss by hydrothermal vent circulation, photoreduction, and hydrolysis. These results inform our understanding of environments that may have been relevant to the formation of life on Earth, as well as processes that could lead to the emergence of life elsewhere in the universe.

## 1. Introduction

One of the greatest scientific mysteries is the origin of life. On Earth, life may have emerged in hydrothermal systems at the bottom of the Hadean ocean [1,2,3,4]. It is hypothesized that this nascent life would have used fixed nitrogen species as a high-potential electron acceptor to oxidize hydrogen and methane and help reduce CO_2_ to organic carbon [5,6,7]. Early metabolisms may have continued to use nitrogen oxides (NO*_x_*) as one source of high-potential electron acceptors [8,9,10]. Other hypotheses for the origin of life place a large emphasis on hydrogen cyanide (HCN) chemistry for protein and nucleotide synthesis [11].

**Figure 1:**
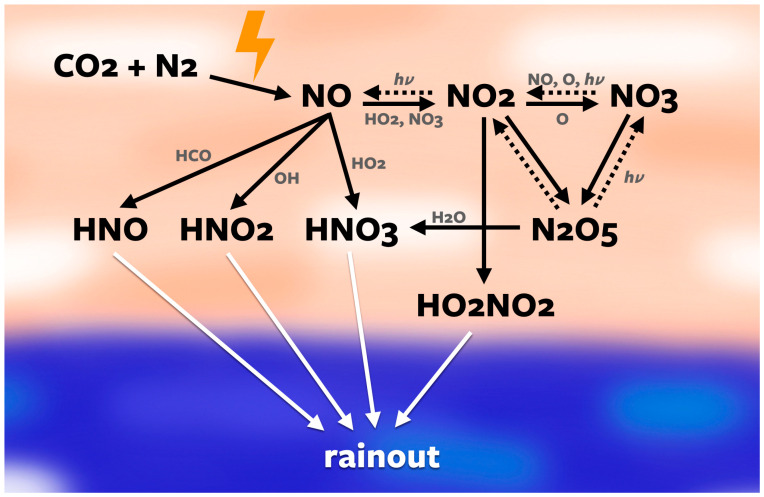
Diagram of the nitrogen oxide reaction pathways. As the pressure and heat of lightning strike these chemicals, it forces them to react and form nitrogen oxides which then react with other species to form nitroxyl (HNO), nitrous acid (HNO_2_), or nitric acid (HNO_3_), which then rain out into the atmosphere.

NO*_x_* can form abiotically on ancient Earth via production in the atmosphere by lightning, which is capable of breaking down CO_2_ and N_2_, followed by photochemistry and rainout to surface waters (Figure 1) [12,13]. Lightning discharges decompose CO_2_ and N_2_ as follows:

CO_2_ → CO + O

O + N_2_ → NO + O

N + CO_2_ → NO + CO.

Because of diatomic nitrogen’s strong triple bond, very few processes are able to decompose N_2_ in the way lightning does. As lightning is discharged into the atmosphere, its current-carrying channel heats the air around it. The shockwave that follows, commonly referred to as thunder, carries that heat to distant particles that dissociate under the heat and pressure of the shockwave. Molecules that form via lightning discharges become kinetically frozen, unable to revert back to their original state because the atmosphere cools back to ambient temperatures in less than a second, kinetically inhibiting reverse reactions [14]. Similarly, HCN is known to form on present-day Earth through impacting solar energetic particles. These break down N_2_ into N and N(^2^D), an excited state of nitrogen, which then react with CH_3_ radicals to form H_2_CN, which then yields HCN [15]. These species are soluble and dissolve in condensed water, which then rains on to the surface.

The atmospheric production and oceanic concentrations of NO*_x_* were estimated for plausible early-Earth conditions by Wong et al. [12]. This study considered lightning production and rainout fluxes to estimate the amount of nitrogen oxides raining out into the ocean, and then only used hydrothermal vent circulation to determine the loss of aqueous nitrogen oxides in the ocean. Adams et al. [16] improved upon the methods of Wong et al. [12] to estimate the concentrations of both nitrogen oxide and hydrogen cyanides on early Mars. The new reactions and processes included in Adams et al.’s [16] study showed that solar energetic particle (SEP) deposition dominates HCN formation in early terrestrial atmospheres, and that photoreduction dominates over hydrothermal circulation as a loss mechanism for aqueous NO*_x_*, as first described by Ranjan et al. [17].

Here, we utilize the modeling framework of Adams et al. [16] and apply it to early Earth to update and expand upon the findings of Wong et al. [12]. We present state-of-the-art estimates of hydrogen cyanide and nitrogen oxides on early Earth to estimate the amount of fixed nitrogen available at the time of life’s emergence and early evolution. We also compute the abundance of carbon monoxide (CO), a molecule that could have added to conducive conditions for the formation of organic molecules and therefore life [18,19].

**Figure 2:**
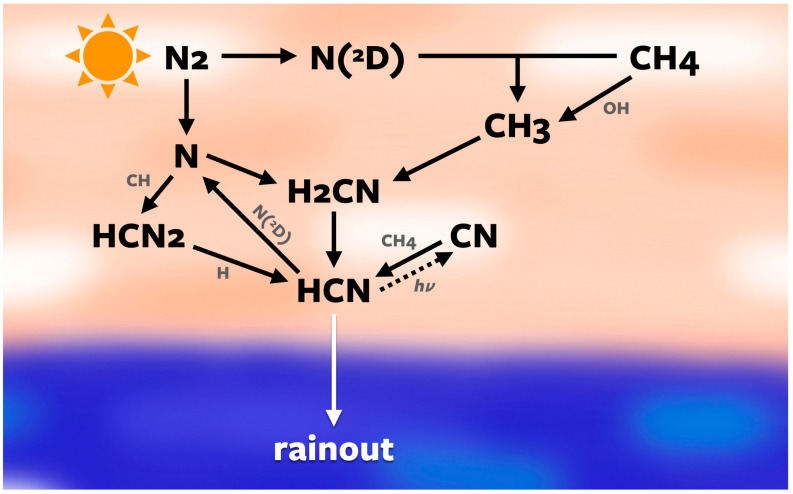
Diagram displaying the hydrogen cyanide pathways. Diatomic nitrogen is broken down into atomic nitrogen, which then reacts with CH_3_ to form methylene amidogen (H_2_CN), which then breaks down via reactions with primarily hydrogen into hydrogen cyanide, which then rains out into the ocean. The left side of the diagram shows the alternate pathway via HCN_2_ added in this study. Some hydrogen photolyzes to CN, which then turns back into hydrogen cyanide through reactions with methane.

Additionally, this study is the first to consider the production of hydrogen cyanide on Earth through a formation pathway involving HCN_2_ (Figure 2), motivated by Berry et al. [20]. These reactions were previously used in Krasnopolsky and Cruikshank [21] to model haze formation on Pluto, but to our knowledge they have never been used in a terrestrial context. To simulate a more accurate and complete atmosphere and estimate of hydrogen cyanide, we have added these reactions to improve estimates of HCN production on early habitable worlds.

## 2. Methods

Due to the vast uncertainty with regard to Hadean atmospheric composition, we modelled 12 different possible Hadean Earth atmospheres. We varied the total pressure between 1 bar, 2 bars, and 12 bars, with background compositions of 90% N_2_, 10% CO_2_ in the 1-bar case, 1 bar N_2_ and 1 bar CO_2_ in the 2-bar case, and 10 bars N_2_ and 2 bars CO_2_ in the 12-bar case. We varied the amounts of H_2_ and CH_4_ in each atmosphere, allowing the surface mixing ratio of each gas to take values of 0.1%, 0.3%, 1%, or 3%. To form HCN and NO*_x_* in an N_2_/CO_2_-dominated atmosphere, an energy source is required. We took into account three sources of energy: lightning, SEPs, and photochemistry.

### 2.1. Thermochemical and Energetic Particle Deposition Modeling

We used the Chemical Equilibrium with Applications (CEA) model [22] to calculate the effect of lightning and its thermal energy (3000 K) and pressure on each of the 12 atmospheric cases. Thermodynamic equilibrium at this immense heat and pressure favors the creation of HCN and NO. The CEA output for HCN and NO was used as input fluxes for the lower boundary conditions in our photochemical model.

SEPs are energetic protons and electrons streaming from the Sun that impact planetary atmospheres. They deposit their energy in the atmosphere and are energetic enough to split the strong triple bond of N_2_. We used the Geant4 simulation platform to run new energy deposition profiles for the atmosphere of early Earth in each of the cases described above. Similar to Adams et al. [16], we assumed a coronal mass ejection (CME) frequency of 1 event per day, as the early Sun would have been much more magnetically active than today, with each event having an average differential energy flux similar to that of the 29 October 2003 CME event [23]. The Geant4 simulation platform computes the flux profiles of N and N(^2^D) produced by SEPs deposited at each altitude layer on early Earth by considering the total SEP energy flux divided by the N_2_ bond-dissociation energy and finally multiplied by the concentration of N_2_ at that altitude. These profiles of reactive N atoms are then included in our photochemical model.

### 2.2. Photochemical Modeling

After computing the lightning-induced fluxes for NO and HCN using CEA and the N and N(^2^D) profiles produced by SEP deposition, we modelled how these species react to produce HNO*_x_* and HCN. To do this, we used the Caltech–JPL photochemistry–transport model KINETICS [24], which solves the continuity equation:

$$\frac{dn_{i}}{dt}=P_{i}-L_{i}-\frac{\partial \varphi_{i}}{dz},$$

where *n_i_* is the number density of species *i*, *φ* is the vertical flux, *P_i_* is the chemical production rate, and *L_i_* is the chemical loss rate evaluated at time *t* and altitude *z*. The vertical flux is given by the following:

$$\varphi_{i}=-\frac{dn_{i}}{dt}\;(D_{i}+K_{zz})-n_{i}(\frac{D_{i}}{H_{i}}+\frac{K_{zz}}{H_{atm}})-n_{i}\frac{\partial T}{dz}[\frac{(1+\alpha_{i})D_{i}+K_{zz}}{dT}],$$

where *D_i_* is the species’ molecular diffusion coefficient, *H_i_* is the species’ scale height, *H_atm_* is the atmospheric scale height, $\alpha_{i}$ is the thermal diffusion coefficient, *K_zz_* is the vertical eddy diffusion coefficient, and *T* is the temperature [25].

Our model considers 495 chemical reactions. We varied the H_2_ and CH_4_ content of the atmosphere, and for each case, the lightning-induced fluxes of the reduced gases computed by CEA were used as lower boundary conditions for NO and HCN in KINETICS. The complete list of boundary conditions in our model is presented in Table 1.

KINETICS then computes the change of species concentration over time, considering both chemical production/loss terms and transport. The model runs for upwards of 10^8^ simulated years to ensure the concentrations converge to a steady state. The outputs of the chemical reactions over time were graphed for analysis (see Section 3).

### 2.3. New Reactions Involving HCN_2_

To create a more realistic and holistic atmospheric reaction network, three reactions were added to the KINETICS database:

CH + N_2_ + M → HCN_2_ + M (R1)

H + HCN_2_ → HCN + NH (R2)

H + HCN_2_ → CH_2_ + N_2_ (R3)

with *k*_1_ = 10^−30^ cm^6^ molecule^−2^ s^−1^, *k*_2_ = 10^−13^ cm^3^ molecule^−1^ s^−1^, *k*_3_ = 10^−12^ cm^3^ molecule^−1^ s^−1^, respectively, and where M denotes background molecules that remove energy from the system. From the lab work of Berry et al. [20] and Trainer et al. [26], it was hypothesized that these reactions would increase the production of HCN in the Hadean Earth’s atmosphere. In this work, we added them to our chemical network and quantified their effect on HCN production (see Section 3.3).

### 2.4. Oceanic Concentrations

As the motivation behind this study is to understand how early life may have formed on Earth, we used the rainout rates of NO*_x_* and HCN, calculated by KINETICS, to estimate oceanic concentrations of NO*_x_* and HCN during the Hadean period. We computed equilibrium concentrations by balancing delivery from the atmosphere with loss due to photoreduction, hydrolysis, and hydrothermal vents, with an assumed Hadean ocean depth of 5.33 × 10^5^ km [27].

Two photoreduction reactions for NO*_x_* were considered:

NO_3_^−^ + *hv* = NO_2_^−^ + ½ O_2_ (R4)

NO_2_^−^ + H_2_O + *hv* = NO + OH + OH^−^ (R5)

with rate constants of *k*_4_ = 2.3 × 10^−8^ s^−1^ and *k*_5_ = 1.2×10^−6^ s^−1^, respectively. These reactions were chosen because they are well constrained and are good representations of the reaction pathway in the ocean [17]. Additionally, when considering loss of HCN within hydrothermal vents, we assumed a water mass of flux through high-temperature vents of 7.2 × 10^12^ kg yr^−1^. We also considered the loss of HCN through hydrolysis, as discussed by Miyakawa et al. [28]. Specifically, the reaction:

$$\mathrm{HCN}\;\overset{H2O,\;k1(HCN)\;}{\to }\;{\mathrm{HCONH}}_{2}\overset{H2O,k1(formamide)}{\to }\;\mathrm{HCOOH}\;+\;{\mathrm{NH}}_{3},$$

where *k*_1_(HCN) and *k*_1_(formamide) are derived from the laboratory hydrolysis rates from the study by Miyakawa et al. [28], which, when fit to an Arrhenius equation, finds a hydrolysis kinetic rate of 2.265 × 10^−12^ molecules/cm^2^/s, corresponding to a temperature of 273 K. These loss processes for NO*_x_* and HCN, combined with our production rates calculated via KINETICS, allowed us to solve for the equilibrium concentrations of these species in the Hadean ocean.

## 3. Results

### 3.1. Lightning-Induced HCN and NO

**Figure 3:**
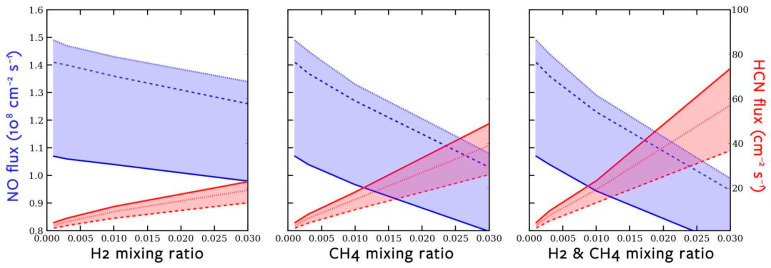
Graph of the CEA results, showing NO and HCN flux over different concentrations of atmospheric composition. Solid, dashed, and dotted lines refer to our 12-bar, 2-bar, and 1-bar cases, respectively. On the left side, H_2_ is the only chemical that increases in concentration, as CH_4_ stays constant at 0.1%, and the same is true for CH_4_ in the middle. On the right, H_2_ and CH_4_ increase together. There is an inverse relationship present between NO and HCN, even though NO is almost six orders of magnitude more prevalent than HCN.

After running the CEA thermochemical model to calculate the creation of NO (the precursor to NO*_x_* species) and HCN by lightning under different background atmospheric compositions, it was clear that NO and HCN respond inversely to increases in background H_2_ and CH_4_ surface mixing ratios (Figure 3). (Note that NO and HCN are on massively different scales of concentration within the atmosphere.) HCN increases as CH_4_ increases, which is to be expected as HCN forms via reactions with CH_3_, a photochemical product of CH_4_. NO decreases as both CH_4_ and H_2_ increase, as both CH_4_ and H_2_ make the environment more chemically reduced, while NO is thermodynamically more favorable in more oxidized environments.

### 3.2. Photochemistry

**Figure 4:**
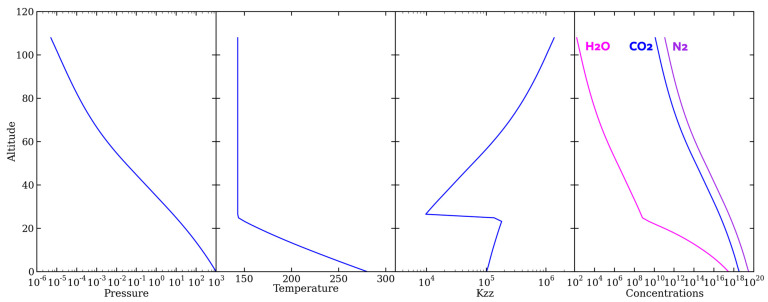
The boundary conditions of KINETICS based on altitude (km) of our 1-bar atmosphere: pressure (mbar); temperature (K); *K*_zz_ or eddy diffusion (cm^2^ s^−1^); and the species concentrations for N_2_, CO_2_, and H_2_O (cm^−3^).

As the photochemical model, KINETICS, runs to a steady state (for over a hundred million simulated years), it computes the amount of each species present in the atmosphere and as a function of altitude. In our 1-bar, 2-bar, and 12-bar atmospheres, we assumed surface temperatures of 280 K, 332 K, and 388 K, respectively, in accordance with the Hadean Earth general circulation model results of Wong et al. [12]. The lower atmospheric temperature profile follows the moist adiabatic lapse rate. Upon reaching a stratospheric temperature of 142.8 K, 139.6 K, or 169 K (again, corresponding to the 1-bar, 2-bar, and 12-bar cases), we set the temperature profile to an isotherm. The eddy diffusion parameter *K_zz_* describes vertical transport within the atmosphere and is computed following the methods of Ackerman and Marley [29]. The H_2_O profile is fixed to the saturation vapor pressure (Figure 4). The common occurrence of carbon monoxide buildup was also seen in this atmosphere (see Section 4.2).

**Figure 5:**
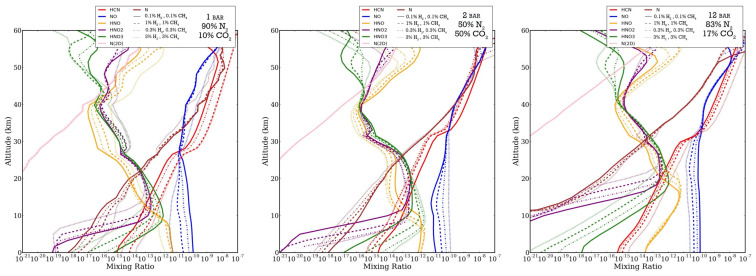
Mixing ratios of NO*_x_*, HCN, HNO, HNO_2_, HNO_3_, N, and N(^2^D) vs. altitude for the (**left**) 1-bar atmosphere, (**middle**) 2-bar atmosphere, and (**right**) 12-bar atmosphere. A different line style presented for each assumed atmospheric concentration. HNO, HNO_2_, and HNO_3_ all tend to mirror each other, and both HCN and NO*_x_* are in relation with their original CEA values.

To effectively compute the concentrations of HCN and NO*_x_*, seven species are the focus throughout the discussion of the results: HCN, NO, HNO, HNO_2_, HNO_3_, N, and N(^2^D) (Figure 5). All of the NO*_x_* and HNO*_x_* species react with and affect the concentrations of each other (Figure 1 and Figure 2). Throughout the atmosphere, the concentrations of HNO, HNO_2_, and HNO_3_ are all similar, with HNO_3_ having slightly lower concentrations than the other two. These species all dissolve in water droplets to rain out on to surface waters, and the equilibrium oceanic concentrations are directly proportional to the rainout rates. N and N(^2^D) vary slightly depending on the atmospheric pressure but are within a consistent range for all starting concentrations.

### 3.3. The Effect of Adding HCN_2_ Reactions

We ran all atmospheric cases with and without the new HCN_2_ reactions to compare the rainout rate of HCN between the atmospheres with and without these reactions and assess the importance of including HCN_2_ pathways in photochemical modeling relevant to the origins of life.

**Figure 6:**
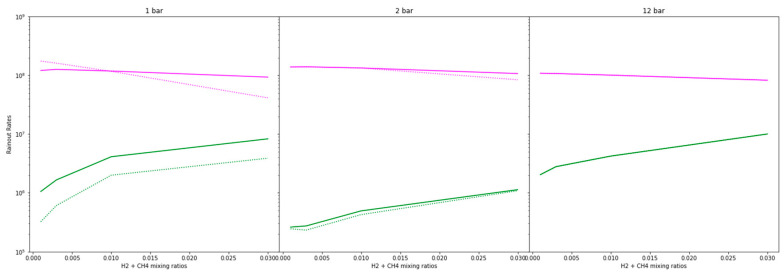
Rainout rates of NO*_x_* (magenta) and HCN (green) for the various atmospheric compositions tested in this study, both with (bold) and without (dashed) the HCN_2_ pathway. The HCN rainout rate rose by a factor of five in the 1-bar case and is about the same in the 2- and 12-bar cases when the HCN_2_ reactions were added. These results present more accurate estimates of how much fixed nitrogen may have been available to early life.

Figure 6 displays the rainout rates, without and with the new reactions, for both NO_x_ (magenta) and HCN (green) per each pressure case. Interestingly, the HCN rainout rate rose by a factor of five in the 1-bar case and is about the same in the 2- and 12-bar cases when the HCN_2_ reactions were added.

### 3.4. Oceanic Concentrations

**Figure 7:**
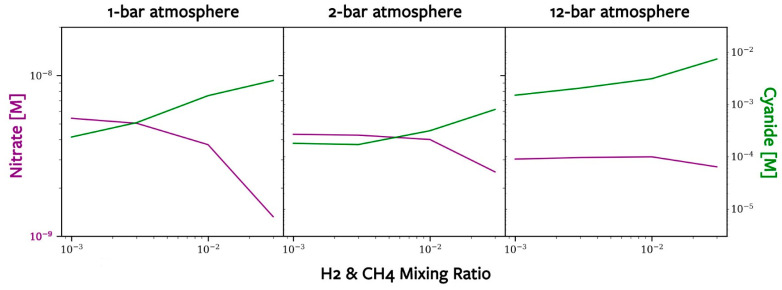
The oceanic concentrations of both NO_3_^−^, in purple, and HCN, in green, in relation to the original atmospheric concentrations of H_2_ and CH_4_. Similar to the original CEA graph (Figure 3), NO_3_^−^ and HCN are in an inverse relationship. Note that the magnitude at which these chemicals are present is completely different, as NO_3_^−^ is <10^−8^ M, whereas HCN is >10^−5^ M.

Once NO*_x_* species and HCN reach the Hadean ocean, they are subject to a number of loss processes. We determined their equilibrium concentrations via the procedure outlined in Section 2.4, and these are shown in Figure 7. An atmosphere with more H_2_ and CH_4_ results in a higher oceanic concentration HCN and a lower ocean concentration of NO*_x_*. This is because N(^2^D) attacking methane acts as a bottleneck for HCN formation: CH_4_ is the source of CH_3_ radicals, which are essential for making H_2_CN and eventually HCN (Figure 2). Additionally, CH_3_ radicals will react with HO*_x_* species, removing HO*_x_* species needed to make NO*_x_* (Figure 1). Therefore, more CH_4_ results in less HO*_x_* and less NO*_x_*. Our sensitivity studies showed that increasing H_2_ alone, on the other hand, does not contribute to CH_3_ radicals, and thus is less impactful on the HCN and NO*_x_* concentrations.

## 4. Discussion

### 4.1. The Relevance of NO_x_ and HCN

We presented updated estimates of NO*_x_* and new estimates of photochemically derived oceanic HCN on early Earth, including the effects of SEP events on both NO*_x_* and HCN and the photoreduction of NO*_x_* in aqueous solution. Additionally, the new HCN_2_ reactions increase the overall amount of HCN in the atmosphere, and hence the HCN rainout into the early Earth’s ocean. This was expected, as these reactions provide an additional pathway of HCN formation, increasing the rainout rate of HCN by a factor of five in the 1-bar case. These results provide an update to the previous results of Wong et al. [12] and Adams et al. [16], and they are compatible with the current scientific narrative of HCN and NO*_x_* being created in the troposphere before raining out and being accessible to any potential life in the ocean.

Our model considered a CO_2_- and N_2_-dominated Hadean atmosphere. Other studies have investigated the nature of the Hadean atmosphere post-giant impact, which causes the atmosphere to take on a much more reduced state. In this post-impact state, Wogan et al. [30] found a slower deposition rate (<1 × 10^5^ HCN/cm^2^/s) than our results for low-CH_4_ (<10%) cases, and Wogan et al. [30] and Pearce et al. [31] found the in high-CH_4_ cases, the HCN production and deposition rates were much larger.

Oceanic concentrations of HCN and NO*_x_* are of astrobiological interest because they are possible sources of fixed nitrogen—a necessity for the formation, maintenance, and evolution of early life. For HCN to be helpful in protein synthesis, local concentrations of at least 0.01 M are required [32]. Our estimated concentrations of NO*_x_* and HCN on early Earth may be helpful to future lab work to determine the habitability, genesity, and urability of the Hadean environment [33,34].

### 4.2. CO Runaway

**Figure 8:**
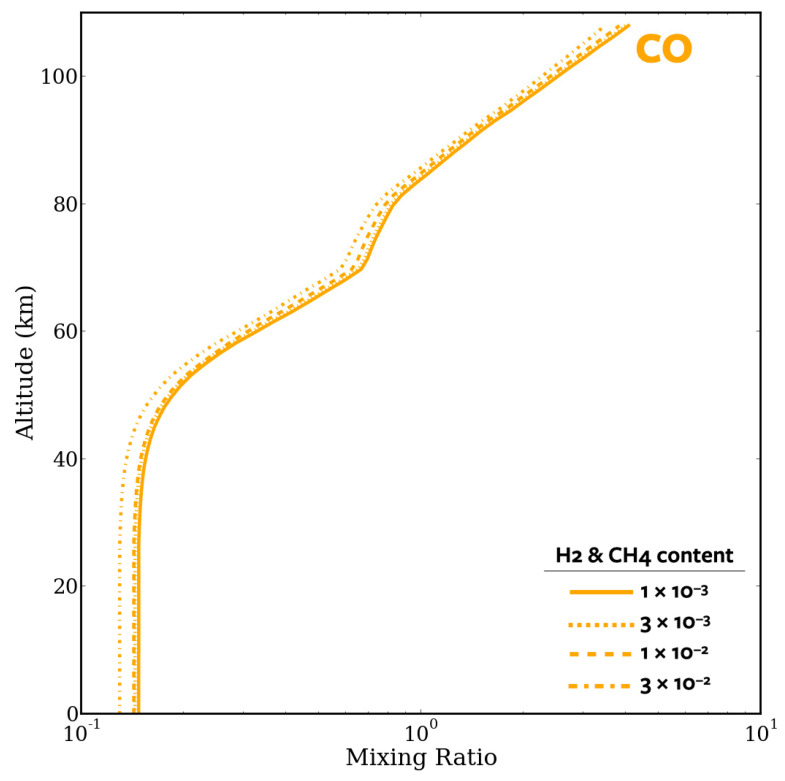
The mixing ratio of carbon monoxide vs. altitude for a 1 bar atmospheric case, where H_2_ and CH_4_ content match. CO is a photochemical product, and the total density is computed from the ideal gas law. While physically a mixing ratio cannot exceed 1, numerically this results in our code due to CO buildup. Since CO_2_ is fixed, whenever CO_2_ is lost to CO_2_ + *hv* → CO + O (and this CO_2_ is not replenished via CO + HO*_x_*), an infinite source of CO_2_ replenishes the profile (numerically). This photolysis therefore occurs over time and CO builds until eventually CO exceeds the total density predicted by the ideal gas law. Again, while we predict the CO buildup is real, its mixing ratio exceeding unity is completely a numerical effect due to fixing CO_2_.

Our models reproduce the previously observed [35] “carbon monoxide (CO) runaway” (Figure 8). Because of the relatively large amount of carbon dioxide present in the Hadean atmosphere, ultraviolet light can photolyze the carbon dioxide, forming atomic oxygen and carbon monoxide. If very little to no water is present at the relevant altitudes, then HO*_x_*, the main family of compounds that reacts with carbon monoxide to cycle it back to carbon dioxide, is unable to react with enough CO to eliminate the runaway via the following process:

CO + OH → CO_2_ + H (R6)

H + O_2_ + M → HO_2_ + M (R7)

O + HO_2_ → O_2_ + OH (R8)

Net: CO + O → CO_2_

As a result, carbon monoxide can accumulate in the atmosphere over time and dominate the composition of the atmosphere. However, after analyzing the model output, it became clear that the lower layer of the atmosphere was not nearly as dominated by CO as the top. This is expected, as the bottom of the atmosphere, where rain forms, has much more water vapor than at the top of the atmosphere.

Based on our modeling, it was possible that an upper-atmosphere CO runaway would be present during the Hadean eon. Although CO does not necessarily build up to a “runaway” state in the lower atmosphere, a significant fraction (over 10%) of the atmosphere is CO in the photochemical steady state across all of our atmospheric cases (Figure 8). This could have profound implications on the creation of organic compounds via CO-related species and pathways [18,19]. Our results motivate further investigation of organic synthesis under high-CO conditions.

We acknowledge that the photolysis rate of water (i.e., production rate of HO*_x_*) is not well constrained (e.g., two largely conflicting rates are presented in Ranjan et al. [36] and Burkholder et al. [37]). Future laboratory work could re-examine H_2_O photolysis rates and compare their results and photolysis rates in practice, allowing the photolysis rates to be more constrained.

### 4.3. Other Considerations

In the future, adding HCN reactions with iron in surface waters, as a loss mechanism to HCN, would add more accuracy to the survival rates and thereby equilibrium concentrations of HCN. Additionally, lab work performed on the NO*_x_* reaction rates with iron would also add immensely to the accuracy of the survival rates of NO*_x_*, as those rates are currently poorly constrained [17].

Further research can build upon the biological context for these new estimates by adding more to our understanding of metabolisms involving nitrate. It is likely that NO*_x_* is important for microbiological metabolisms and the origin of life, but it is unknown how much NO*_x_* is needed for this to be important. Recent work [38] has shown that the precursor molecules pyruvate and nitrate can be converted into the amino acid glutamine via reductive amination on ferroan brucite, a common mineral in hydrothermal vent systems, but these system effects have not been analyzed as a full system. Clarification of these reactions and processes would help determine the potential for the emergence of life in these oceanic environments.

In conclusion, the additional HCN_2_ reactions (R1–R3) and updated atmospheric processes added here improve upon HCN and NO*_x_* rainout estimates from that of past research. The common “problem” of a CO runaway is present in our model, but only dominates the upper, thinner part of the atmosphere. A high-CO lower atmosphere may be of interest to future work on prebiotic chemistry. Further lab work on this as well as widening information on chemical networks and microbial metabolisms could help us eventually reach the goal of understanding the roots of life on early Earth and, potentially, early Mars and Earth-like exoplanets.

## 5. Conclusions

Understanding the processes of nitrogen fixation remains one of the keys to understanding the emergence of life on Earth and other planets. We estimated Hadean NO and HCN lightning-induced fluxes of approximately 10^8^ and a few to ~70 cm^−2^s^−1^, respectively, by computing the thermochemical equilibrium with the CEA model. We computed Hadean N and N(^2^D) flux profiles based on solar energetic particle events using the Geant4 simulation platform. These were used as inputs for our photochemical model, the Caltech–JPL model KINETICS, which we used to derive the Hadean precipitation rates of HNO*_x_* and HCN (on the order of 10^8^ molecules/cm^2^/s and 3 × 10^5^–1 × 10^7^ molecules/cm^2^/s, respectively). With the assumed losses to photodestruction and hydrothermal vent circulation, we found concentrations of nitrate of about 1 × 10^−9^–6 × 10^−9^ M and hydrogen cyanide of about 1 × 10^−2^–1 × 10^−4^ M. We suggest more laboratory research to fully understand the astrobiological implications of these concentrations.

## Figures and Tables

**Table 1:** The boundary conditions used in this study. If a range is shown, that value varies between atmospheric cases. If the number is in bold, it represents a deposition velocity (cm s^−1^); standard text, a fixed flux (molecules cm^−2^ s^−1^); and italicized, a fixed mixing ratio. The HCN and NO*_x_* ranges displayed are referenced from Figure 3.

Species	Lower Boundary	Upper Boundary
O	0	0
O_2_	**−1.00 × 10^−6^**	0
O_3_	**−1.00 × 10^−2^**	0
H_2_	*1.00 × 10^−3^*– *3.00 × 10^−2^*	0
HO*_x_*	0	0
N*_x_*H*_y_*	**−1.00 × 10^−2^**	0
HNO*_x_*	**−1.00 × 10^−2^**	0
N*_x_*O*_y_*	0	0
CH*_x_* (*x* ≠ 4)	0	0
CH_4_	*1.00 × 10^−3^*– *3.00 × 10^−2^*	0
CO	**−1.00 × 10^−6^**	0
N	0	−1.60 × 10^9^
H*_x_*C*_y_*O	**−1.00 × 10^−2^**	0
NO	1.07 × 10^8^– 1.49 × 10^8^	0
HCN	3.43 × 10^0^– 6.67 × 10^0^	0
C*_x_*H*_y_* (*y* = even)	**−1.00 × 10^−3^**	0
C*_x_*H*_y_* (*y* = odd)	**0**	0
CN	**−1.00 × 10^−2^**	0
CxN	**−1.00 × 10^−2^**	0
H*_x_*C*_y_*N*_z_*	**−1.00 × 10^−2^**	0

## Data Availability

This study was a theoretical study and the models we used are appropriately referenced.
